# Impact of pre-annealing process on electrical properties and stability of indium zinc oxide thin-film transistors

**DOI:** 10.1038/s41598-022-24093-w

**Published:** 2022-11-14

**Authors:** Han-Lin Zhao, Gergely Tarsoly, Fei Shan, Xiao-Lin Wang, Jae-Yun Lee, Yong Jin Jeong, Sung-Jin Kim

**Affiliations:** 1 College of Electrical and Computer Engineering, Chungbuk National University, Cheongju, 28644 Korea; 2 Department of Materials Science and Engineering, Korea National University of Transportation, Chungju, 27469 Republic of Korea

**Keywords:** Materials science, Nanoscience and technology

## Abstract

This paper examined the effects of no treatment versus plasma treatment, and femtosecond laser irradiation as pre-annealing processes on indium zinc oxide (IZO) films and annealing at high temperatures. The plasma pre-annealed multilayer stacked IZO TFTs showed better electrical properties with mobility enhancement from 2.45 to 7.81 cm^2^/Vs, but exhibited diminished on–off current ratio (I_on_/I_off_). The IZO thin-film transistor (TFT) prepared with femtosecond laser pre-annealing with low pulse energy generation (power of 3 W at 700 nm wavelength) for 100 s has also exhibited significantly improved electrical performance, the saturation mobility increased to 4.91 cm^2^/Vs, the I_on_/I_off_ ratio was enhanced from 4.5 × 10^5^ to 2.1 × 10^6^, the threshold voltage improved from − 1.44 to − 0.25 V, and the subthreshold swing was reduced from 1.21 to 0.61 V/dec. In conclusion, IZO TFTs with improved performance can be prepared using a femtosecond laser pre-annealing process, which has great potential for fabricating low-cost, high-performance devices.

## Introduction

Amorphous metal oxide semiconductors have been studied extensively for the development of thin-film transistors (TFTs) because of their applicability in wearable electronic circuits, favorable optical transparency in the visible region, and reduced fabrication costs through low-temperature processes. Indium oxide, indium zinc oxide (IZO), indium gallium zinc oxide, and several other metal oxides have been utilized^1–6^.

IZO is one of the widely used active layer materials for TFTs because of its wide bandgap (3.68–3.76 eV), high mobility, high transparency, and smooth surface^7–10^. Multi-stacked IZO layers have lower porosity and higher density than single layers and can generate more electron carriers and exhibit improved electrical properties. Multi-stacked IZO films need to be annealed several times during preparation to obtain high-density films^11,12^. Multi-stacked IZO TFTs can be fabricated using solution processes, vacuum evaporation, chemical vapor deposition, and atomic layer deposition techniques. Of these, solution processes have the advantages of simple fabrication, low-temperature processing, and low cost^13–19^. In addition, oxygen (O_2_) plasma treatment, photochemical activation, femtosecond laser treatment, and thermal annealing under an O_2_ atmosphere were shown to improve the surface quality of the films and enhance the electrical properties of multi-stacked IZO TFTs^20–22^. The plasma pre-annealing treatment at low power (< 150 W) was demonstrated to improve the electrical properties of oxide TFTs by reducing the charge carrier concentration and conductivity, and offers the advantages of low cost, high efficiency, and large area uniformity^23,24^. A pre-annealing process using femtosecond lasers applies the laser to a specific spot of the film, resulting in less thermal damage in the film through which the laser passes. Femtosecond laser technology is used widely for TFT preparation because of its relatively short laser pulses and high transient intensity, which reduces the cost and avoids damage to oxide films^25,26^.

In this study, TFTs composed of IZO films treated with different pre-annealing processes of plasma and femtosecond laser were fabricated to achieve high mobility and relatively good electrical stability and provide a relatively simple, low-cost preparation method. In addition, the effects of different pre-annealing processes on IZO TFTs were compared.

## Experimental details

**Figure 1:**
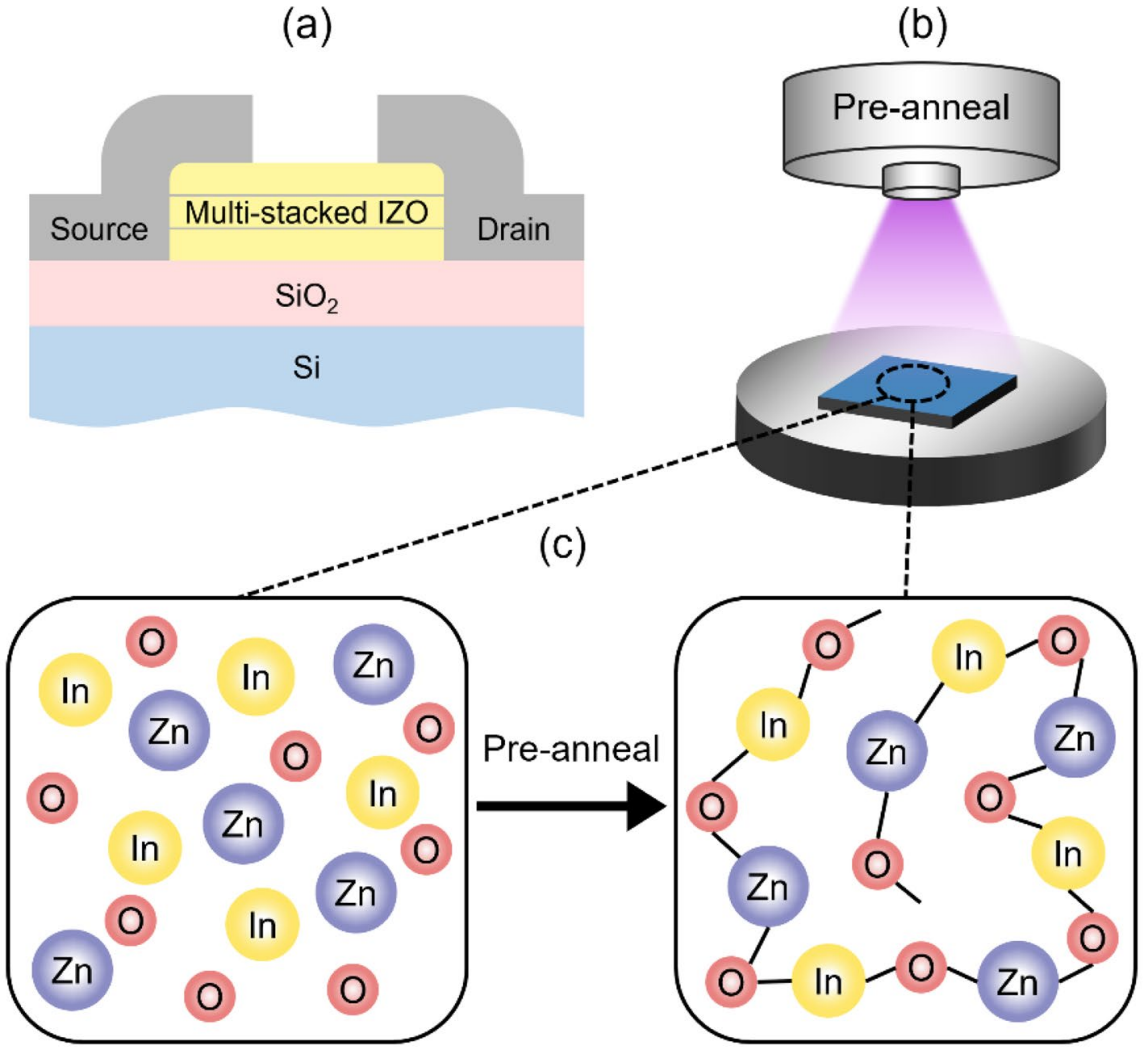
(**a**) Schematic diagram of the device fabrication process for multi-stacked IZO TFTs. Sketches of (**b**) plasma and (**c**) femtosecond laser pre-annealing.

A multi-stacked IZO film was manufactured on the a heavily dopped n-type Silicon bottom gate substrate that has a hot-grown 100 nm thick Silicon dioxide layer used a dielectric layer, as shown in Fig. 1a. First, the substrate was cleaned with piranha solution (3:1 ratio of sulfuric acid to hydrogen peroxide) at 80 °C, sonicated sequentially in DI water, acetone, and isopropanol for 20 min each at 45 °C, and blow dried with nitrogen gas. All residual solvent was removed by drying the samples in an oven at 60 °C for 30 min.

2.5 ml of a 0.1 M precursor solution of indium nitrate hydrate [In(NO_3_)_3_·xH_2_O] in 2-methoxyethanol was prepared in a glass vial, and 50 μl of acetylacetone, and 22.5 μl of ammonia were added. Subsequently, 1.5 ml of a 0.1 M zinc acetate dihydrate [Zn(CH_3_COO)_2_·2H_2_O] solution in 2-methoxyethanol was prepared, and 30 μl acetylacetone was added. Both solutions were placed in a magnetic stirrer at 700 rpm and stirred at 60 °C for 1 h. The two solutions were mixed at a 1:1 ratio and stirred at 500 rpm and room temperature for 2 h to prepare the IZO precursor solution. The mixed solution was filtered through a 0.2 μm syringe filter to remove particulates and to obtain a more transparent and homogeneous solution, which is beneficial for spin-coating. The precursor solution was spin-coated on four clean silicon substrates at 1,500 rpm for 30 s, followed by a 30 min annealing process at 400 °C. This resulted in an IZO thickness of 20 nm. The same spin-coating and annealing processes were repeated to form the second layer. For forming the third layer, the spin-coating process under the same conditions was repeated again, but with different pre-annealing conditions. To fabricate the devices with plasma treated layers, the IZO layer was treated with oxygen plasma at an RF power level of 150 W for 3 min (with the schematics shown in Fig. 1b], where the plasma produced a uniform O_2_^+^/e^−^ radiation over an appropriate area, transforming from O_2_ at 3 sccm. For the laser irradiated IZO films, an ultrashort pulse mode-locked titanium:sapphire femtosecond laser system (Coherent, Chameleon Ultra II) generated at low pulse energy with a power of 3 W and a 700 nm wavelength was used for 100 s. The schematics of the effect of pre-annealing is shown in Fig. 1c. For control, samples with neither laser nor oxygen plasma pre-annealing treatment were also prepared. All devices (treated and untreated) were hard baked for 1 h at 400 °C, and then cooled back to room temperature. The 100 nm thick aluminum source and drain electrodes were deposited on top of the IZO films by thermal evaporation (pressure ~ 10 − 6 Torr, rate 0.1 Å/s) through the same shadow mask with a channel width of 2000 μm and a length of 200 μm.

The surface morphology and properties of multi-stacked IZO films were analyzed by atomic force microscopy (AFM) and X-ray photoelectron spectroscopy (XPS). The electrical characteristics of the output, transfer, and stability of the TFTs were measured using a semiconductor parameter analyzer (Keithley 4200, Keithley Instruments LLC, Cleveland, Ohio) in a dark room at room temperature.

## Results and discussion

**Figure 2:**
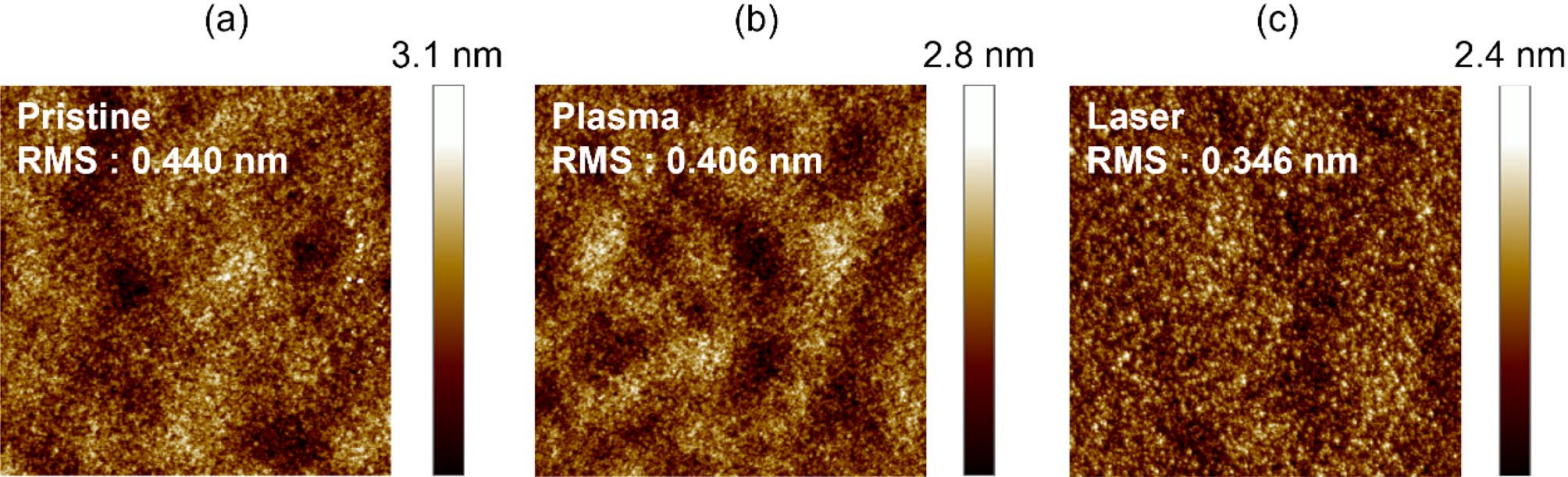
AFM images of multi-stacked IZO thin-film surfaces under (**a**) pristine, (**b**) plasma, and (**c**) femtosecond laser pre-annealing.

The surface morphology of the multi-stacked IZO films treated with different pre-annealing methods was analyzed by AFM; Fig. 2a–c show the corresponding height distribution of each AFM image. The root mean square (RMS) roughness of pristine, plasma, and femtosecond laser pre-annealed IZO films was 0.440, 0.406, and 0.346 nm, respectively. From the cross-sectional image, plasma pre-annealing positively affects the surface roughness of the multi-stacked IZO films. The film surface was smoother and more uniform than the pristine sample. The IZO films irradiated with femtosecond laser pre-annealing showed a more uniform granular structure, and the deposited films showed a continuous and smooth surface with no obvious wave crests. The roughness may contribute to the leakage currents, which deteriorate the electrical properties (e.g., electron mobility) of the device^27,28^. This suggests that a proper pre-annealing process involving plasma or femtosecond laser treatments can lead to more homogeneous multi-stacked IZO films.

**Figure 3:**
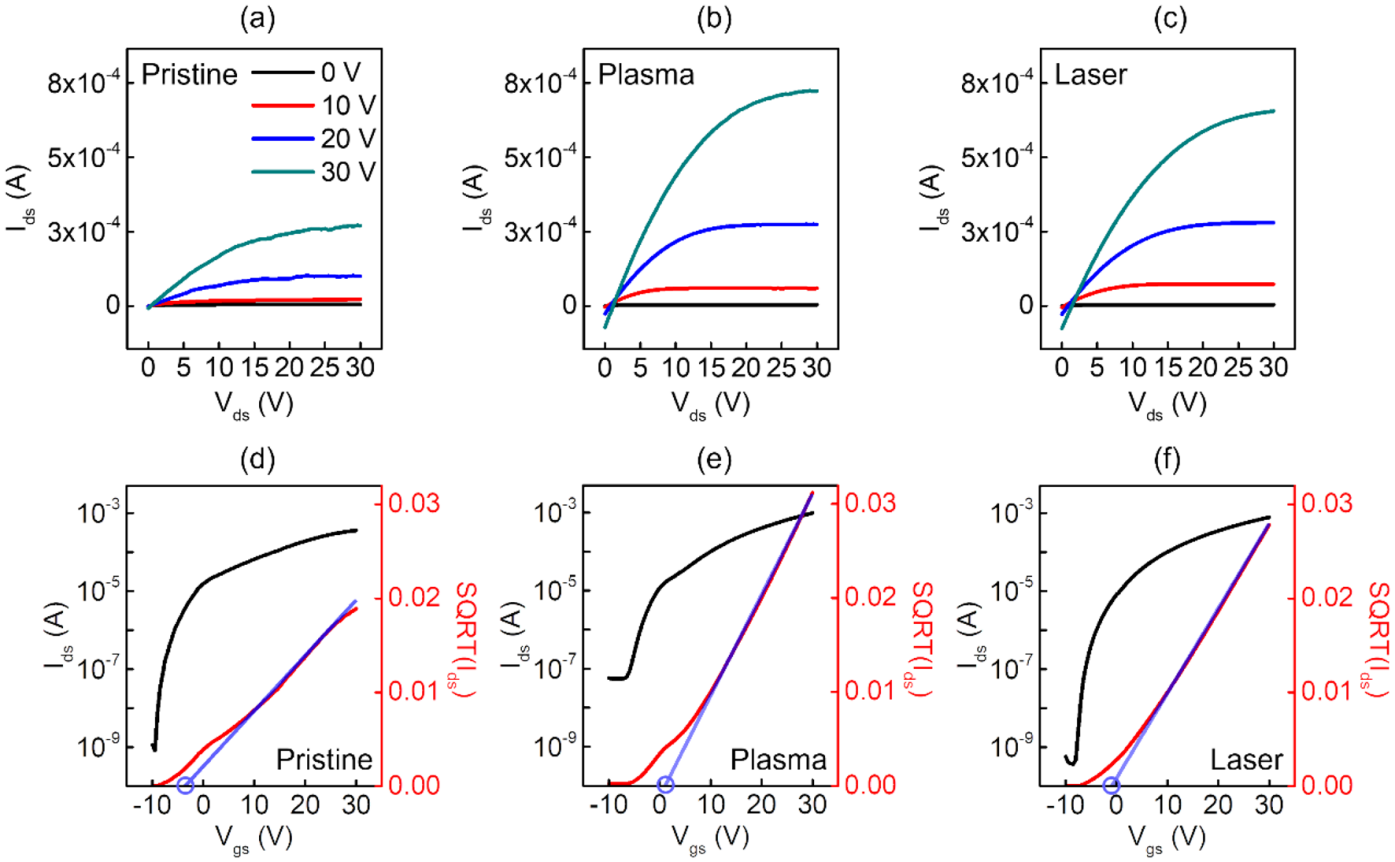
Output characteristics of multi-stacked IZO TFTs with (**a**) pristine, (**b**) plasma, and (**c**) femtosecond laser pre-annealing. Transfer characteristics and SQRT transfer curves of multi-stack IZO TFTs with (**d**) pristine, (**e**) plasma, and (**f**) femtosecond laser pre-annealing.

Figure 3a–c show the output characteristic curves of the drain current (I_ds_) at constant gate bias voltages (V_gs_) of 0, 10, 20, and 30 V, drain bias voltage (V_ds_) incremented from 0 to 30 V in 10 V increments to investigate the electrical characteristics of the trhee types of IZO TFTs. All devices exhibited typical *n*-type output characteristics, but the one with femtosecond laser and plasma treated IZO had smoother and more stable saturation curves than the other IZO TFTs. On the other hand, the femtosecond laser and plasma treated IZO TFTs showed leakage currents at low drain voltages. Figure 3d–f shows the transfer curves, and square root (SQRT) transfer curves of the pristine, plasma, and femtosecond laser pre-annealing treated TFTs when the bias voltage was applied with V_ds_ fixed at 30 V as V_gs_ was increased from − 10 V to 30 V in 0.5 V increments. All the TFTs showed typical n-type characteristics as I_ds_ increased with V_gs_ and exhibited a significant transition from the off-state to the on-state.

**Figure 4:**
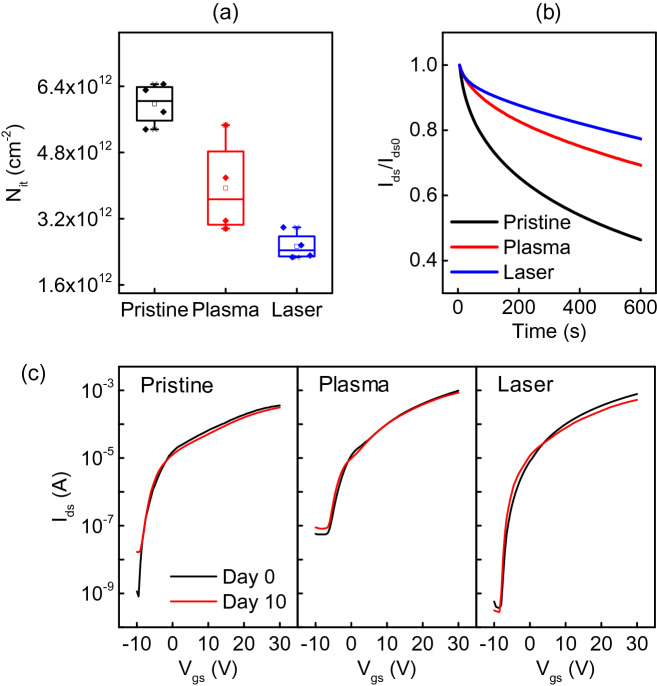
Multi-stacked IZO TFTs (**a**) interface trap charges, (**b**) stress time stability characteristics in pristine, plasma, and femtosecond laser pre-annealing and (**c**) transfer curves of devices compared after 10 days of storage under ambient conditions.

For comparison, Table 1 lists the saturation mobility (μ_sat_), threshold voltage (V_th_), on/off current ratios (I_on_/I_off_), and subthreshold swing (SS) values of the pristine, plasma, and femtosecond laser pre-annealed multi-stacked IZO TFTs. Compared to the device with pristine IZO, which had a mobility of μ_sat_ = 2.45 cm^2^/Vs, the devices with plasma treatment had higher electron mobility (μ_sat_ = 7.81 cm^2^/Vs).The ones with the femtosecond laser treatment also showed improved performance (μ_sat_ = 4.91 cm^2^/Vs). On the other hand, the I_on_/I_off_ of the plasma-treated IZO TFT decreased compared to the pristine one, with V_th_ shifting from − 1.44 V to 1.13 V in the positive direction. By contrast, the femtosecond laser treatment resulted in an increase in I_on_/I_off_ to 2.1 × 10^6^, and an improved V_th_ shift to − 0.25 V. Significant improvements in the SS values were also observed for both pre-annealed IZO TFTs, particularly for the femtosecond laser-treated ones, from the 1.21 V/dec of the pristine device to 0.61 V/dec. The improved electrical properties of the devices correlated with the lower SS values, which relate to the interfacial trap density (N_it_), as expressed in Eq. (1),

1 $${\text{N}}_{{{\text{it}}}} = \frac{{{\text{C}}_{{\text{i}}} }}{{\text{q}}}\left[ {\frac{{{\text{SSlog}}\left( {\text{e}} \right)}}{{{\text{kT}}/{\text{q}}}} - 1} \right],$$

where C_i_ is the accumulation capacitance of an insulator per unit area; q is the electron charge, k is the Boltzmann’s constant, and T is the temperature. The calculated N_it_ values of the pristine, plasma, and femtosecond laser-treated multi-stacked IZO TFTs were 5.98 ± 0.43, 3.94 ± 0.96, and 2.53 ± 0.29 (× 10^12^ cm^−2^), respectively (Fig. 4a). The interfacial trapping density is closely related to the SS, and a decrease in the SS value signifies a decrease in the number of traps at the interface^29,30^. The use of femtosecond laser results in the smallest number of interface trap states among the three types of devices, resulting in better overall electrical performance when measuring the characteristics of multilayer IZO TFTs.

**Table 1:** Electrical performance of multi-stack IZO TFTs with pristine, plasma, and laser pre-annealing.

Treatment methods	μ_sat_ (cm^2^/Vs)	I_on_/I_off_	V_th_ (V)	SS (V/dec)	N_it_ (× 10^12^ cm^-2^)
Pristine	2.45	4.4 × 10^5^	− 1.44	1.21	5.98 ± 0.43
Plasma	7.81	1.8 × 10^4^	1.13	0.68	3.94 ± 0.96
Laser	4.91	2.1 × 10^6^	− 0.25	0.61	2.53 ± 0.29

The bias stress stability characteristics of the pristine, plasma, and femtosecond laser-treated TFTs were measured. The time evolution of I_ds_ measured in the TFTs normalized to the initial value with a duration of 600 s and V_gs_ and V_ds_ both biased at 30 V (on state), as shown in Fig. 4b. The decrease in current with stress time was evident, with an initial rapid decrease followed by a prolonged and slow decrease without establishing a steady state. This behavior is consistent with a shift in the device V_th_ caused by charge trapping^31^. The pristine IZO surface exhibited the highest attenuation of the drain current due to the presence of many defects, with the normalized I_ds_ dropping to a final value of 0.77 after 600 s. The normalized I_ds_ of the plasma treated devices has dropped to 0.69, and for the femtosecond laser one it decreased to 0.77 during the same time interval. The femtosecond laser irradiated devices show improved stability. On the long term, the device may deteriorate due to environmental factors such as water, oxygen, etc. Measuring the device performance 10 days after the fabrication, for the pristine device, a tenfold increase in the off curent was observed, but other parts of the transfer curve has remained relatively unchanged, as shown in Fig. 4c. For the devices with either plasma or laser pre-annealing treatment, there is no significant change in the transfer curves. These results demonstrate that the pre-annealing treatments have some passivation effect on semiconductor thin film.

**Figure 5:**
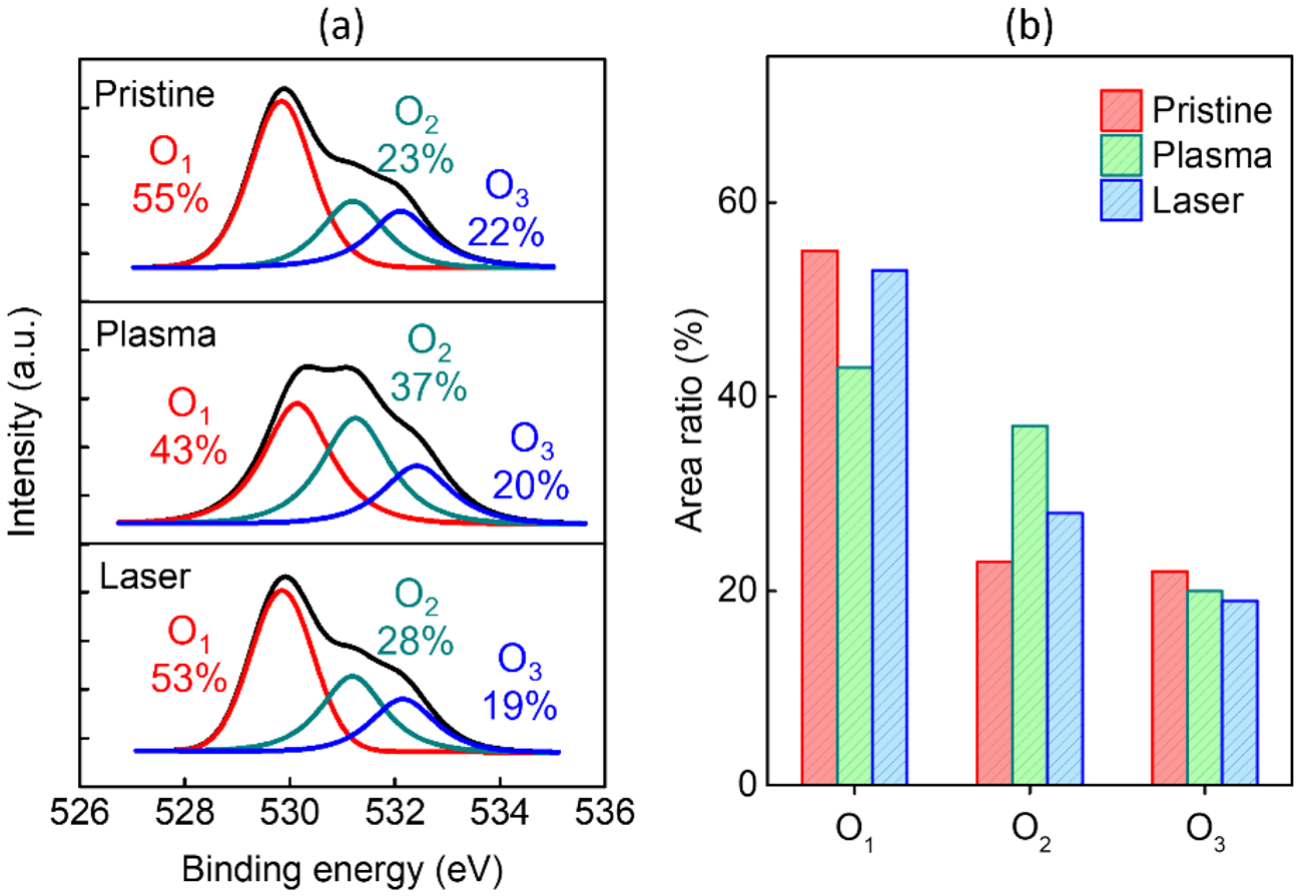
(**a**) XPS O 1s analysis of multi-stacked IZO films with pristine, plasma, and femtosecond laser pre-annealing. (**b**) Analytical areas of O1 peaks for M–O lattices, O_V_ states, and O–H species based on different pre-annealing processes of pristine, plasma, femtosecond laser.

The effect of oxygen content of the IZO films on the electrical properties of the devices was studied. The multilayer structured IZO films were examined by XPS to determine the effects of pre-annealing methods of plasma and femtosecond laser treatment on the IZO films compared to the pristine sample device, as shown in Fig. 5a. The O 1s peak was fitted by three near-Gaussian curves centered at 530.1 (O_1_), 531.2 (O_2_), and 532.4 (O_3_) eV, which were assigned to metal-bonded oxygen (M–O), oxygen vacancies (O_V_), and hydroxide species (O–H), respectively. Figure 5b shows the O 1s area ratios of the three Gaussians. The plasma pre-annealed IZO films had a higher O_V_ peak area, but the area of M–O peak decreased. O_V_ is generally considered to be a shallow donor that is formed by the breakage of In–O and Zn–O bonds. Compared to the pristine IZO TFTs, the oxygen atmosphere in plasma pre-annealing promoted the decomposition of M–O, increasing the O_V_ and improving the carrier concentration, and affecting the associated mobility. The relative area of the M–O lattice peaks of the femtosecond laser pre-annealed films were reduced relative to the original, and the area of the O_V_ peaks was increased^25,32^. The variation of the electrical properties is consistent with the trends in Table 1 and Fig. 5b. The proportion of O–H groups was similar, resulting in a minor effect on the electrical performance.

## Conclusion

The effects of plasma, and femtosecond laser pre-annealing processes on the performance of multi-stacked IZO-based devices were studied by comparing the characteristics to devices with pristine multi-stack IZO active layers. The plasma pre-annealed multi-stacked IZO TFTs showed improved electrical properties with a mobility of 7.81 cm^2^/Vs. The optimized preparation process for the femtosecond laser pre-annealed IZO TFTs was demonstrated, showing an improved I_on_/I_off_ enhancement to 2.1 × 10^6^. Femtosecond laser pre-annealing has important theoretical and practical implications for improving the electrical properties and time stress stability of oxide IZO TFTs for the low-cost fabrication of amorphous oxide electronics.

## Data Availability

The dataset used and/or analyzed during the current study available from the corresponding author on reasonable request.
